# Low-intensity transcranial focused ultrasound neuromodulation of the amygdala: is left always right?

**DOI:** 10.1038/s41380-025-03319-z

**Published:** 2025-10-27

**Authors:** Annakarina Mundorf, Sebastian Ocklenburg

**Affiliations:** 1 ISM Institute for Systems Medicine and Department of Human Medicine, MSH Medical School Hamburg, Hamburg, Germany; 2 ICAN Institute for Cognitive and Affective Neuroscience and Department of Psychology, MSH Medical School Hamburg, Hamburg, Germany; 3 Biopsychology, Institute for Cognitive Neuroscience, Faculty of Psychology, Ruhr University Bochum, Bochum, Germany

**Keywords:** Psychiatric disorders, Neuroscience

With great interest, we read the recent study by Barksdale et al., 2025 [1] investigating low-intensity transcranial focused ultrasound (tFUS) neuromodulation targeting the left amygdala in a double-blind, sham-controlled treatment trial for mood, anxiety, and trauma-related disorders. This is a timely and impressive contribution to the growing field of non-invasive brain stimulation, and the authors are to be commended for the technical precision and clinical ambition of their approach.

While employing a technically advanced methodology, the study’s fixed approach of stimulating only the left amygdala may limit treatment responsiveness in some patients due to interindividual differences in brain lateralization. Based on previous literature, developing a protocol to determine the stimulation site individually for each patient may optimize the efficacy of the treatment in individual patients. This could be achieved by assessing individual brain lateralization using methods such as handedness questionnaires combined with functional imaging techniques like fMRI or functional transcranial Doppler sonography (fTCDS), comparing activity between hemispheres. For deep structures such as the amygdala, fMRI remains the best method to assess laterality. However, EEG or fTCDS during emotion processing tasks or behavioral tasks like emotional dichotic listening can provide a relatively rapid and cost-efficient general estimate of a person’s hemispheric lateralization [2–4], which may still be useful in guiding initial treatment decisions. Considering the variability and complexity of lateralization, these assessments can help tailor stimulation to each patient’s unique brain organization.

The decision to target the left amygdala was motivated by prior evidence suggesting more prominent left-sided abnormalities in certain psychiatric populations, e.g., anxiety patients [5], using a protocol previously shown to attenuate left amygdala activation during fear provocation [6]. Interestingly, while the current study justifiably focused on the left amygdala due to evidence of more prominent left-sided abnormalities, the inclusion of earlier right-sided stimulation protocols [7] highlights the potential relevance of both hemispheres in tFUS research and suggests that a more balanced or individualized targeting strategy may be beneficial in future studies.

That said, we noted that the study did not include information on participants’ handedness, nor any structural or functional markers of hemispheric lateralization. While understandable given the exploratory focus of the study, such measures are increasingly recognized as crucial in psychiatric neuroscience, as many brain structures, including the amygdala, display robust left-right differences that influence both cognition and emotion [8–11]. Including these factors in future studies will enhance our understanding of individual variability in treatment response.

Specifically, the left and the right amygdala are not functionally equivalent, and stimulating them could yield differential treatment effects [10, 11]. For example, the amygdala shows several forms of functional lateralization: the left is more involved in conscious emotional evaluation, while the right is more engaged in unconscious threat detection and rapid emotional responses [10]. Regarding fear processing, the right amygdala is linked to sensory fear responses, while the left is linked to cognitive fear learning and extinction [11]. Moreover, the right amygdala has a pro-nociceptive function in pain processing, while the left amygdala has an anti-nociceptive function [12].

While these population-level asymmetries are empirically well-supported, they are only part of the story. The literature also contains some inconsistencies, further emphasizing the importance of individualized assessments. Typically, most patients within a cohort exhibit asymmetry consistent with the population norm; however, some individuals show a reversed asymmetry, which contrasts with the population average. For these individuals, stimulation of the left amygdala would have similar effects to stimulating the right amygdala in most of the population.

The absence of such asymmetry measures in neuromodulation studies may obscure meaningful inter-individual variability and reduce the generalizability of findings, particularly in clinical populations known to deviate from typical lateralization patterns [13–15]. This complexity underscores the value of assessing lateralization markers, such as handedness, in neuromodulation research. While handedness primarily reflects motor dominance, it has been associated with broader patterns of hemispheric specialization and can thus provide a useful first indicator of potential lateralization differences, particularly given the higher variability observed in left-handers. For example, atypical language lateralization occurs in approximately 30% of left-handed individuals, compared to only about 5% of right-handers. Functional asymmetries for non-verbal domains, such as visuospatial attention, manual praxis, and body perception, also tend to be more variable in left-handers, with right-hemisphere dominance for body perception in 94% of right-handers but only 70% of left-handers [16]. Importantly, non-right-handedness, i.e., left- and mixed-handedness, is more frequently observed in clinical populations [17], further supporting the relevance of handedness assessment in clinical neuromodulation studies. Its omission in the current study represents a missed opportunity to investigate potential laterality-dependent effects of tFUS. Incorporating such measures could facilitate more nuanced post-hoc analyses and help identify subgroups who may benefit from targeted stimulation of the contralateral amygdala [18].

As the field of neuromodulatory treatments advances, the need for personalized approaches is becoming increasingly evident. Research in transcranial magnetic stimulation has shown that treatment efficacy relies heavily on aligning stimulation with an individual’s unique brain connectivity patterns [19]. This variability in brain connectivity supports the growing rationale for personalized neuromodulatory treatments, where interventions are tailored to each patient’s specific brain network architecture and hemispheric lateralization. Similarly, interindividual variation in structural and functional brain asymmetry is substantial, even among healthy individuals [17]. In clinical populations, this variation becomes especially important, as psychiatric and neurological disorders often involve atypical brain lateralization, which can influence both symptom expression and treatment response [17]. Incorporating asymmetry metrics into future treatment protocols could improve precision medicine by aligning neuromodulatory treatments with each patient’s distinct neurobiological profile. Pre-treatment lateralization mapping using fMRI, fTCDS, or EEG during emotional tasks may offer similar advantages for ultrasound-based interventions, allowing tFUS protocols to be tailored to individual profiles, potentially improving treatment responsiveness and reducing non-responder rates.

In the prior pilot study targeting the right amygdala in individuals with generalized anxiety disorder, sixteen out of twenty-five participants reported meaningful clinical improvement at treatment completion [7]. Similarly, the current study demonstrated a moderate-to-large treatment effect on symptom outcome (Cohen’s *d* = 0.77) [1] following left amygdala stimulation. However, not all participants responded to treatment in either trial, suggesting that variability in individual brain lateralization could influence therapeutic outcomes. Most neurofeedback research has focused on up-regulating the left amygdala to enhance emotion regulation [20], whereas findings on right amygdala down-regulation [21] provide complementary evidence of functional lateralization. These data collectively underscore the potential benefits of incorporating pre-treatment assessments of hemispheric lateralization to refine target selection and further enhance clinical efficacy by aligning stimulation with each patient’s dominant emotional processing circuits.

Finally, to optimize the efficacy and interpretability of neuromodulation studies, future research should actively embrace the ethical and clinical value of accounting for individual differences in hemispheric specialization. Stimulating a non-optimal hemisphere, particularly in patients with atypical lateralization, may reduce treatment efficacy or obscure true effects. To enhance treatment precision of non-invasive neuromodulation tools as they transition into clinical practice, it is relevant to incorporate lateralization-sensitive measures, pre-treatment neuroimaging, or even bilateral targeting protocols. Given that emotional and cognitive symptoms may be differentially modulated depending on which hemisphere is targeted, accounting for these factors will not only improve treatment precision but also deepen our understanding of the neurobiological mechanisms underlying psychiatric disorders.
